# Suitability of Biomorphic Silicon Carbide Ceramics as Drug Delivery Systems against Bacterial Biofilms

**DOI:** 10.1155/2013/104529

**Published:** 2013-07-07

**Authors:** P. Díaz-Rodríguez, A. Pérez-Estévez, R. Seoane, P. González, J. Serra, M. Landin

**Affiliations:** ^1^Departamento de Farmacia y Tecnología Farmacéutica, Facultad de Farmacia, Universidad de Santiago de Compostela, 15782 Santiago de Compostela, Spain; ^2^Departamento de Microbiología, Facultad de Medicina, Universidad de Santiago de Compostela, 15705 Santiago de Compostela, Spain; ^3^Departamento de Física Aplicada, E.E. Industriais, Universidade de Vigo, 36301 Vigo, Spain

## Abstract

The present work is aimed at getting a new insight into biomorphic silicon carbides (bioSiCs) as bone replacement materials. BioSiCs from a variety of precursors were produced, characterized, and loaded with a broad-spectrum antibiotic. The capacity of loaded bioSiCs for preventing and/or treating preformed *S. aureus* biofilms has been studied. The differences in precursor characteristics are maintained after the ceramic production process. All bioSiCs allow the loading process by capillarity, giving loaded materials with drug release profiles dependent on their microstructure. The amount of antibiotic released in liquid medium during the first six hours depends on bioSiC porosity, but it could exceed the minimum inhibitory concentration of *Staphylococcus aureus*, for all the materials studied, thus preventing the proliferation of bacteria. Differences in the external surface and the number and size of open external pores of bioSiCs contribute towards the variations in the effect against bacteria when experiments are carried out using solid media. The internal structure and surface properties of all the systems seem to facilitate the therapeutic activity of the antibiotic on the preformed biofilms, reducing the number of viable bacteria present in the biofilm compared to controls.

## 1. Introduction

The pathogenic events taking place on the surface of medical devices are primarily associated with the presence of microorganisms and their biofilms [1, 2]. A biofilm is an intricate community of microorganisms embedded in a polysaccharide matrix, capable of attaching onto different kinds of surfaces developing a hard-to-eradicate infection [3]. The adhesion of bacteria onto a surface (biological or artificial) depends on biophysical properties, such as wettability and/or electrostatic forces, and the production of specific factors such as polysaccharide intercellular adhesins that create links between the bacteria themselves and bacteria surface. Microorganisms reach the implanted medical devices during or immediately after orthopedic surgery, thus leading to further complications [4]. Among postoperative problems, infections caused by *S. aureus* arise from the worst prognosis the ability of this microorganism to adhere to foreign bodies forming biofilms. The formation of biofilms is a key part in antibiotic resistance [5].

Strategies have been developed to prevent biofilm formation after surgery by surface modification of biomaterials which in turn should modify the bacterial adherence [6] or the load and release of broad-spectrum antibiotics from the biomaterials, thus eliminating the incipient colonization [7, 8]. When antibiotics are used, they can be embedded, absorbed into the material structure, or adsorbed on to the biomaterial surface [7, 8]. The antibiotic release from the biomaterial must be sufficient to maintain the local concentration above the minimal inhibitory concentration (MIC) value during a sufficient period of time [9–12]. Several studies aimed at the prevention of colonization and biofilm formation in biomaterials for implants have been reported [13]. While postoperative osteomyelitis is still an important problem in orthopedic and dental clinical practice [14], studies on already formed biofilm treatments are much more limited.

Biomorphic silicon carbide (bioSiC) is a ceramic material obtained from natural resources with good mechanical properties, high biocompatibility, and osteoconductivity [15–17]. BioSiCs have a smart hierarchical porous microstructure (pore size distribution, pore orientation, and total porosity) widely determined by the material used as wood cellulosic preform. In addition, the molten silicon infiltration, characteristic of its manufacturing process, produces a material with a close to the bone Young's modulus [18, 19]. On this basis, bioSiC has been proposed as a candidate material for the production of bone substitutes able to prevent the loss of bone characteristic of other implants made with materials of greater strength [20] and a porous microstructure adequate to load and release antibiotics [16].

In a previous paper we have demonstrated the capacity of bioSiC from sapelli wood to load and release vancomycin, inhibiting bacterial adherence and preventing biofilm formation [16]. The present work aims at extending this previous study to get an insight into new utilities of bioSiCs as bone replacement materials. To the best of our knowledge the suitability of biomorphic silicon carbides to treat already formed biofilms has not been verified yet. We have included bioSiCs from a variety of precursors and therefore different surface and microstructural characteristics, in order to establish any possible differences in its behavior, when the use of these antibiotic loaded bioSiCs for preventing or treating *S. aureus* biofilms is intended.

## 2. Materials and Methods

### 2.1. Bioderived Silicon Carbide

Disks of bioSiC (*Ø*6 mm × 2 mm) from wood precursors with different microstructures were obtained, pine (*Pinus pinaster*), oak (*Quercus robur*), and sapelli (*Entandrophragma cylindricum*), as previously reported by González and coworkers [15]. The wood was dried at 60°C during 24 hours, followed by pyrolysis at 1000°C in nitrogen atmosphere. The carbon preform obtained was then infiltrated with molten silicon in vacuum at 1550°C for 30 minutes.

### 2.2. BioSiC Characterization

The material density was determined, in triplicate, using a helium-air pycnometer (Quantachrome Mod. PY2, USA).

The pore size distribution was evaluated by mercury intrusion porosimetry (Micromeritics AutoPore IV 9500, Norcross, GA, USA) using a 3 mL penetrometer for solids.

The specific surface area was evaluated by adsorption of nitrogen using the Brunauer-Emmett-Teller (BET) method. The disks were degassed by heating at 60°C and 10^−3^ mm Hg. Samples were exposed to N_2_ gas at 77 K and 0.01–0.98 relative pressure using an automatic surface area analyzer (Micromeritics ASAP 2000, USA). BioSiC disks morphology was characterized by Scanning Electron Microscopy (SEM Philips XL 30).

### 2.3. Vancomycin Loading

Vancomycin solutions (42.5 and 85 mg/mL) were prepared by direct dissolution of vancomycin hydrochloride (Fagron Bach: 06L2101) in ultrapure water. Fixed volumes of each solution (30 *μ*L) were added on to the disks. Drug-loaded disks were dried at 40°C until a constant weight was reached.

### 2.4. Vancomycin Release in Dissolution Medium

Dried drug-loaded disks were transferred to vials containing 1 mL of phosphate buffer saline (PBS) pH 7.4 at 37°C and maintained under mechanical shaking. Release medium samples were withdrawn at regular intervals. The vancomycin concentration was evaluated spectrophotometrically at 280 nm (Agilent 8453, Germany).

### 2.5. Vancomycin Elution on Agar Plates *In Vitro*

*Staphylococcus aureus* ATCC 292135 was purchased from the Spanish Collection of Type Cultures (CECT), cultured in brain infusion broth (Liofilchem, Italy) overnight at 37°C in atmospheric conditions, adjusted to 0.5 McFarland units, and used to inoculate Mueller-Hinton Agar (MHA) plates. Immediately, dried vancomycin loaded disks were centered on the inoculated MHA plates, incubated for 24 hours aerobically at 37°C, and then the inhibition halos were measured. Once the halos were measured, the disks were transferred to freshly inoculated MHA plates, as reported before. This procedure was repeated every 24 h until absence of inhibition. 

We have analyzed the release of vancomycin from the three types of samples in different media in order to compare the behavior of different bioSiCs in loading and releasing antibiotics and to confirm their utility in preventing *S. aureus* growth and also treating already formed *S. aureus* biofilm.

### 2.6. *S. aureus* Biofilm Formation and Antibiofilm Activity of Loaded BioSiCs

Biofilms of *S. aureus* were induced on cellulose nitrate membrane filters according to that described by other authors with some protocol modifications [21, 22]. Aliquots (15 *μ*L) of an overnight culture of *S. aureus* grown in brain heart infusion broth (0.5 McFarland) were seeded onto cellulose nitrate membrane filters (13.0 mm diameter, 0.22 *μ*m pore diameter; Millipore, USA) previously situated on MHA plates. Seeded membrane filters on MHA plates were incubated for 1 day at 37°C in atmospheric conditions. Biofilms of *S. aureus* were induced.

Dried bioSiC-vancomycin disks (dose = 2.54 mg) were placed in the center of the membranes containing the biofilm and incubated at 37°C. Unloaded bioSiC disks (control_1_) and sterile paper disks impregnated with 20 *μ*L of a standard vancomycin solution used in microbiological studies (control_2_) (1.5 mg/mL) were used as controls. After incubation for 24 or 48 hours, the dried bioSiC-vancomycin disks and controls were carefully removed, and the treated biofilms were then washed with 5 mL of phosphate buffer saline (PBS) (pH 7.4) to eliminate nonadherent cells, finally transferred to a vial containing 5 mL of PBS (pH 7.4), and vigorously vortexed for 1 minute to suspend adhered cells. The cell suspensions obtained were 10-fold diluted in PBS. Aliquots (50 *μ*L) of each dilution were seeded onto MHA plates, and the colony forming units (CFUs) were counted after 48 hours of incubation at 37°C in atmospheric conditions. This protocol minimized residual activity of the antibiotic. Alternatively, induced biofilms, treated with loaded bioSiCs, and controls were directly studied after gold coating using SEM (Zeiss EVO LS 15, Germany).

### 2.7. Statistical Analysis

Results are expressed as means and standard deviations. Statistical significant differences between treatments were evaluated by analysis of variance (ANOVA) and Fisher's Least Significant Difference (LSD) using Statgraphics X64 software. 

## 3. Results

### 3.1. BioSiC Characterization

Trees are classified into two main groups, softwoods and hardwoods. For the study we have included three wood materials, one softwood, pine, and two hardwoods, oak and sapelli.

**Figure 1:**
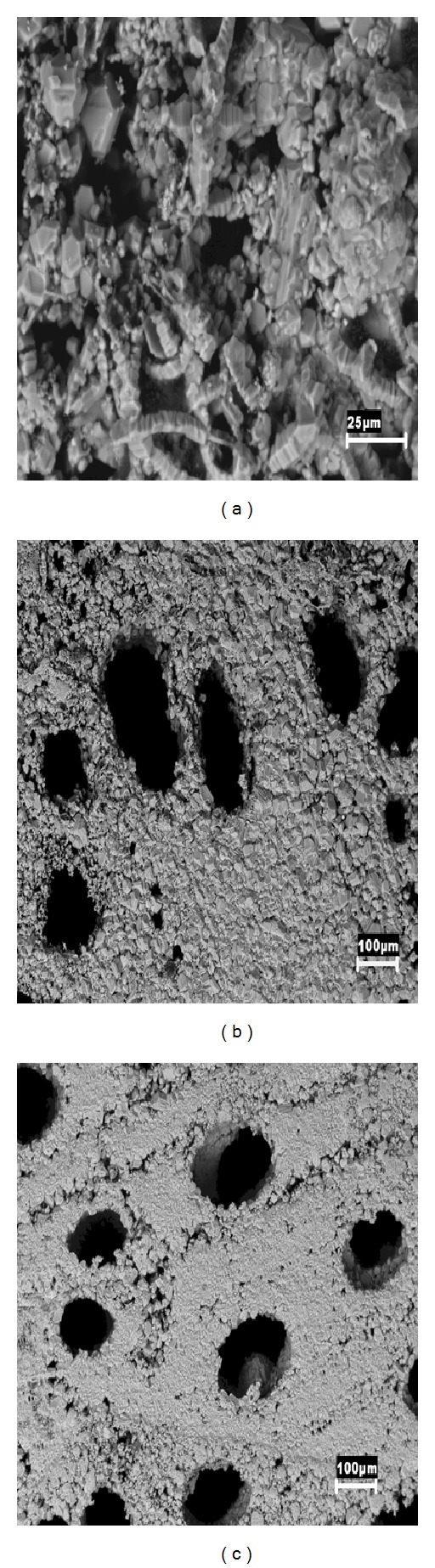
Transverse surface of different bioSiC pieces characterized by Scanning Electron Microscopy (SEM): (a) pine bioSiC, (b) oak bioSiC, and (c) sapelli bioSiC.

Morphological characterization of bioSiC from those different precursors was carried out by SEM micrographs of the transverse surface of material pieces (Figure 1). The major difference between the anatomy of hardwoods and softwoods is the lack of vessels in softwood which are substituted in this type of tree by smaller tracheids (5–50 *μ*m) to conduct the fluid in the trunk. This anatomical peculiarity is the origin of the variations in mechanical properties between softwood and hardwood. As it can be seen, the variations in the internal structures and distribution of vessels, fibers, and rays of the precursors can be still detected after the infiltration of molten silicon. BioSiCs show structures extremely dependent of the source material. Pine bioSiC shows a roughness surface with small external pores while oak and sapelli bioSiCs present open external pores bigger than 100 *μ*m. 

**Figure 2:**
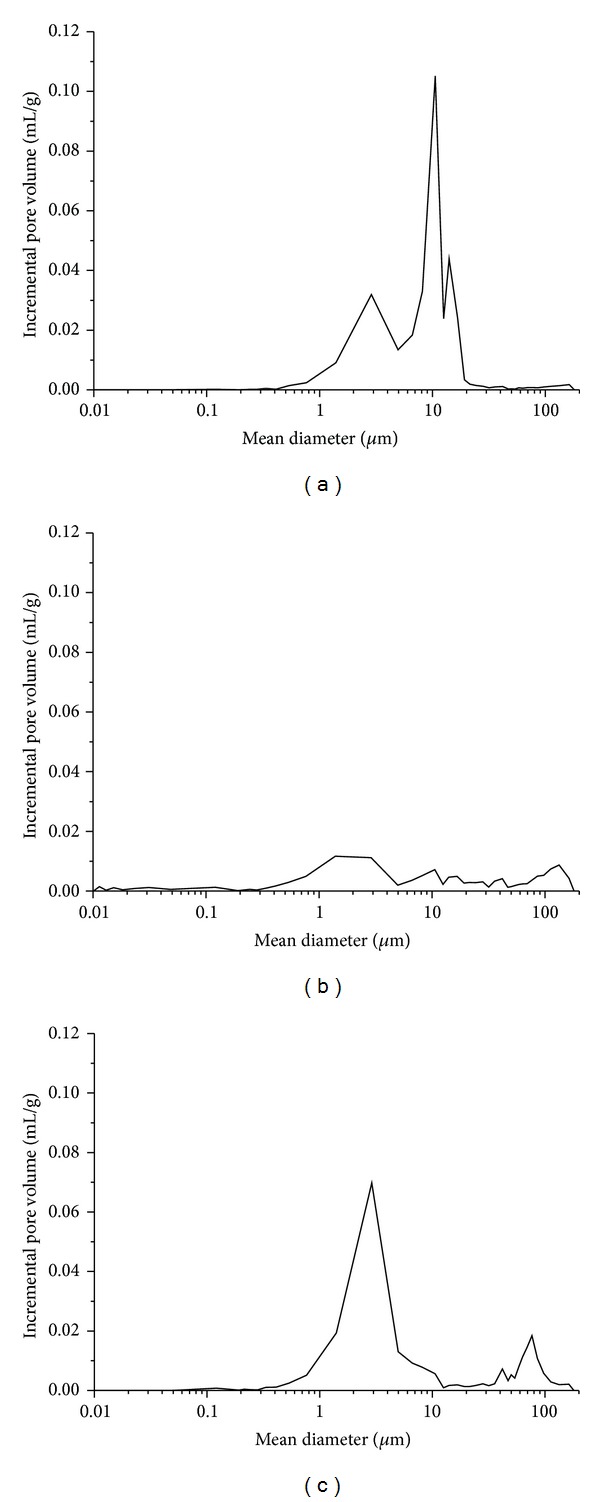
Pore size distribution obtained from mercury intrusion porosimetry of bioSiC of (a) pine, (b) oak, and (c) sapelli.

Mercury intrusion porosimetry results corroborate those observations (Figure 2 and Table 1) and also point out differences between the hardwoods selected. Pine wood gives the material with the highest porosity (46.97% ± 5.43) characterized by numerous interconnected mesopores in the range 1–10 microns (Figure 2(a)).

Oak wood results in the bioSiC of the lowest total porosity (27.85% ± 2.99) and density and the highest specific surface characterized by a bimodal pore distribution including the presence of an important number of macropores (Figure 2(b)) (mean diameter 141 ± 35 *μ*m).

Sapelli bioSiC results (Figure 2(c)) are characteristic of a high porous material (40.72% ± 1.06) with a bimodal pore size distribution with macropores (mean diameter 88 ± 31 *μ*m) and mesopores (mean diameter 3.1 ± 1.6 *μ*m). The agreement between the sapelli bioSiC outcomes is consistent with our previous results in this material [16] pointing out the robustness in bioSiC production process.

The nitrogen adsorption analysis confirmed the absence of microporosity in the bioSiCs. Those variations in the bioSiC surfaces, microstructures, and porosities should result in important variations in behavior with regard to their osteointegration and vascularization properties [23] and also in their capacity to load and release antibiotics.

### 3.2. Vancomycin Release Kinetics

The microstructural characteristics and wettability properties of the bioSiCs allowed all of them to be loaded with a known amount of drug by simply adding the vancomycin solution on to the disks which completely penetrates and maintains within the materials by capillarity.

**Figure 3:**
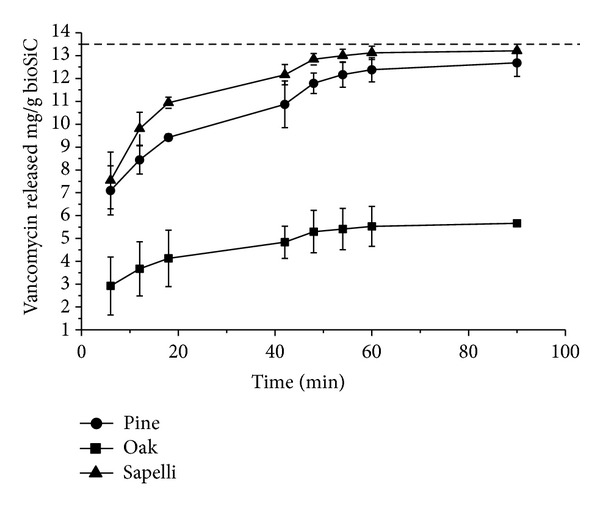
Release profiles of the low load concentrations of vancomycin from three types of bioSiCs from different precursors in PBS. The dashed lines (-) indicate the doses of vancomycin and correspond to 100% drug released.

When the loaded disks were immersed in 3 mL phosphate buffer pH 7.4 in order to simulate the release process, a rapid delivery occurred during the first 90 minutes followed by a slower rate after for all the materials.  Figure 3 shows the profiles for the first hours (a critical period after surgery). The high hydrosolubility of vancomycin (>100 mg/mL) justifies the rapid initial delivery that should correspond to the adsorbed antibiotic on external surface, and the drug molecules with shorter diffusion pathway cause the release of vancomycin from the disks which continues for days.

The vancomycin release kinetics was analyzed using the Higuchi model (Table 2) that can accurately describe the release of water soluble drugs incorporated in porous solid matrices and allow materials to be compared [24]. The good fit of release profiles to this model during the first stage indicates a characteristic diffusion mechanism of the drug through the full medium pores in the bioSiC disks. Differences in total porosity justify the slower release of vancomycin and lower values of *K*_*H*_ (Higuchi dissolution constant) observed for loaded oak disks.

For longer periods, the differences in the antibiotic release profiles in PBS medium from different materials can no longer be observed. The results do not show statistically significant differences between materials for amount drug released at 6 hours. 

The amount of antibiotic released during the first six hours would exceed the minimum inhibitory concentration 90% of *Staphylococcus aureus* set to 1 *μ*g/mL [22] even dipping disks in 1 liter of dissolution, whatever the type of wood precursor used.

### 3.3. Drug Elution in Agar Plates *In Vitro*

**Figure 4:**
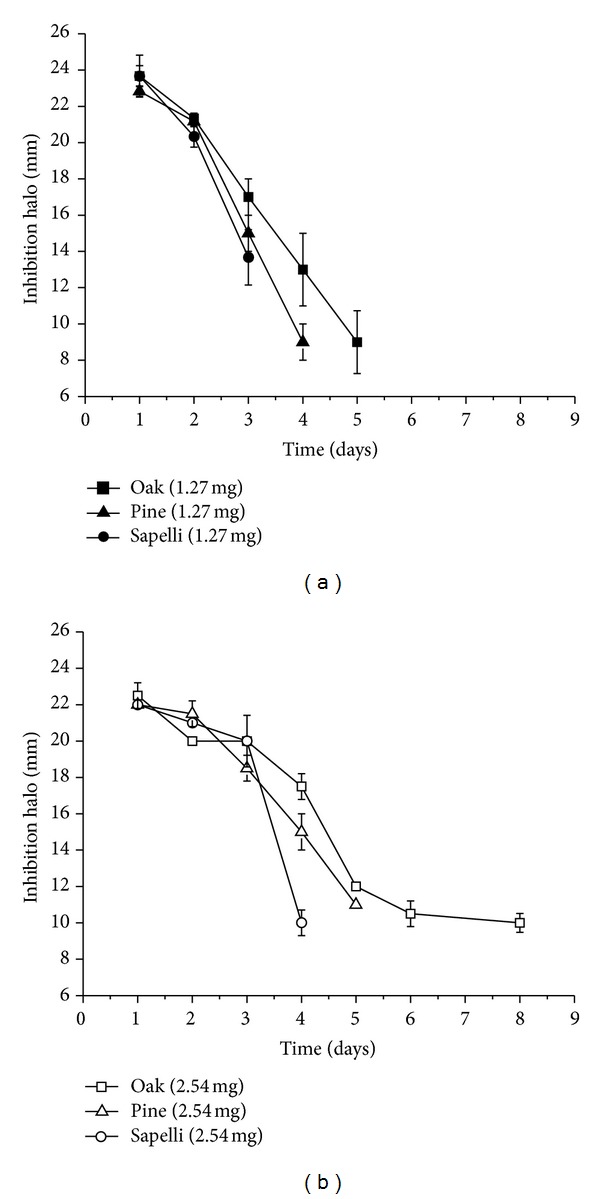
Inhibition halo profiles obtained from and *Staphylococcus aureus* culture for the treatment with different vancomycin doses 1.27 mg and 2.54 mg loaded bioSiCs.

The marked differences in vancomycin release profiles in liquid medium are reflected in the pattern of the inhibition halos of *S. aureus* generated by loaded bioSiC disks (1.27 mg) in cultures on agar (Figure 4(a)). No statistical differences were found between the inhibition halo sizes of the three porous structures at the beginning of the experiment. The drug release rate is a critical factor that determines the time while the device system manages to overcome the minimum inhibitory concentration and therefore generate a measurable inhibition halo. Presumably, the amount of vancomycin transferred to the agar medium should depend also on the number of water molecules available to dissolve the drug and the external surface characteristics of the material. The lowest external surface of oak bioSiC and its big open pores in contact with the agar medium contribute to explain the prolonged effect against bacteria found for this material.

Loaded sapelli, pine, and oak bioSiCs show no bacterial growth inhibitory effect after 3, 4, and 5 days of incubation, respectively. It is possible to improve antibacterial activity against *S. aureus* by increasing the loaded vancomycin dose to 2.54 mg (Figure 4(b)) achieving 4, 5, and 8 days for sapelli, pine, and oak bioSiCs respectively.

### 3.4. Antibiofilm Activity

Table 3 shows the number of the CFUs counted after 24 and 48 hours of treatment of *S. aureus* biofilms previously formed with the different loaded bioSiC systems (Dose = 2.54 mg) and controls. The gray scale illustrates the statistically significant differences between groups. It is interesting to note that control_2_ (standard solution of vancomycin), included as control at just 24 hours of treatment obtained a number of CFUs lower than those of control_1_ (unloaded bioSiC) but significantly higher than CFU after loaded bioSiC treatments. The sustained vancomycin release from all bioSiCs significantly reduces *S. aureus* biofilms indicating that the surface roughness and porous structure of the material could favor the penetration and the slow diffusion of drug through the glycocalyx matrix formed by bacterial population, improving biofilm treatments. The CFUs of *S. aureus* on oak bioSiC at 24 and 48 h were slightly higher than for pine and sapelli bioSiCs. However, results on agar (Figure 4(b)) showed a longer antibacterial effect on oak bioSiC (8 days for loaded oak bioSiC with 2.54 mg of vancomycin). This longer release should be enough to eradicate the infection and could explain the higher CFUs at 24 and 48 h.

**Figure 5:**
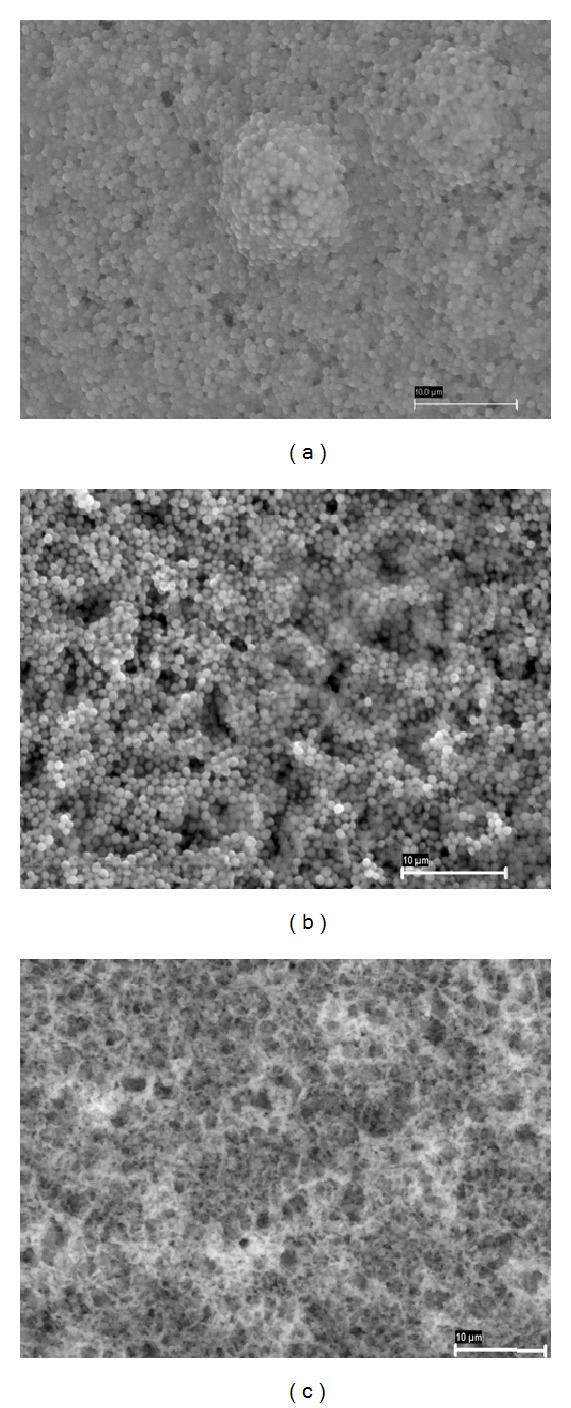
SEM micrographs of (a) induced biofilm on cellulose nitrate membrane, (b) induced biofilm treated with unloaded oak bioSiC (control_1_) after 48 h incubation, and (c) induced biofilm treated with vancomycin loaded (2.54 mg) oak bioSiC after 48 h incubation.

As an example, SEM micrographs of the *S. aureus* induced biofilms together with the biofilms before and after 48 h of treatment with the oak loaded and unloaded bioSiCs (Figure 5). As we can see, the amount of bacteria after 48 h in contact with the unloaded bioSiC (control_1_) (Figure 5(b)) is similar to the nontreated biofilms (Figure 5(a)) and clearly higher than biofilm treatments with loaded bioSiC disks (Figure 5(c)) whose surface appearance becomes clean as the original cellulose nitrate membrane.

## 4. Discussion

Wood is a natural material of complex hierarchical structure as a result of the orientation and alignment of cells that may serve as hierarchical template to generate novel biomorphic ceramics with meso- and macrostructures depending on the precursor selected. The morphology and arrangement of the different cells may vary widely between the different kinds of wood, with large vessel cells dominating in hardwood and tracheids dominating in softwood. The diameter of the vessels and tracheids (named as pores) varies between 5 and 50 *μ*m in softwood and between 1 and 300 *μ*m in hardwood. While pine wood produces ceramics with a homogeneous porous structure characterized by the presence of pores of small size, probably difficult to be colonized by cells, sapelli and oak produce ceramics with interesting porous structures which can be used as implants [25]. The procedure in this work for producing bioSiCs from different natural resources allows systems with variable porosity, specific surface, and roughness to be obtained. The characteristic cells of softwood and hardwood with a preferential orientation in the axial direction offer the possibility of transforming the bioorganic wood structure into an inorganic ceramic material with tailored physical and mechanical properties. Their surface characteristics and internal microstructure make them interesting candidates as potential vectors of therapeutic molecules [16]. 

Local administration of antibiotics through those porous systems would favor therapeutic success, achieving a high dose of drug at the implant site and simultaneously reducing the adverse effects of systemic administration.

The vancomycin release study in PBS for all materials shows a quick antibiotic delivery during the first hours after implantation, characteristic of a high water soluble drug followed by a slow but prolonged release for a number of days. The high initial drug release could act as an attack dose in response to the high risk of infection during the initial shock, and the later controlled drug release keeps antibiotic concentration above the minimum inhibitory concentration (MIC), obtaining an extended antimicrobial therapeutic effect, preventing biofilm formation, and inhibiting the occurrence of latent infections [26]. Differences in drug release kinetics were found regarding the precursor materials, oak bioSiC having lowest porosity and the slower antibiotic release rate. As a consequence, loaded oak bioSiC ceramics extend residual antimicrobial activity longer than pine and sapelli when drug elution was tested on solid medium such as agar. An increase in the loaded dose, from 1.27 mg to 2.54 mg, vancomycin, improves the effectiveness of the treatment, which in the case of the bioSiC from oak extends the prevention of biofilm formation for a week in solid medium. The amount of water molecules and the drug concentration gradient at the prosthesis interface should also affect the antibacterial activity of loaded bioSiCs. Considering the physiological conditions in a bone-implant interface after surgery, where an inflammatory process is present and the inevitable antibiotic clearance due to blood and lymphatic stream has taken place, we could expect the drug release to be higher *in vivo* than the one observed on agar. However, even with this slow drug release, obtained inhibition halos suggest the therapeutic potential of these systems.

After surgery a competition between cells and bacteria for the implant colonization is established [26]. The release of vancomycin locally from implants would favor osteoblast colonization while avoiding bacterial adhesion to the surface and therefore preventing the formation of biofilm. The formation of this organized structure confers resistance to antibiotics, hampers the therapeutic success of treatments, and can lead to severe complications, such as destruction of local tissues, patient disability and morbidity, and sometimes death [27]. The biofilm hinders the penetration of drugs throughout [22, 28, 29]. In this situation the antibiotic has poor activity against biofilm-embedded bacteria promoting resistances as a consequence of the continuing exposure to low drug concentrations [30].

The high molecular weight of vancomycin [31], the possible inhibition reactions with exopolysaccharides of the matrix, and others factors could be responsible for the slow diffusion of this drug through biofilms. As a result, the MIC_90_ of vancomycin is sharply increased in bacteria biofilms from 1 to 8 *μ*g/mL [32]. Our results indicate that vancomycin released from the bioSiCs was enough to treat *S. aureus* biofilms, progressively decreasing the number of viable bacterial cells embedded on the structured matrices. The rough structure of the scaffolds facilitates the antibiotic penetration through. On this basis, vancomycin loaded bioSiCs could be considered potential candidates to produce implants for the substitution of infected prostheses in chronic infections reducing the risk of relapse.

## 5. Conclusions

There are statistically significant differences in surface characteristics, density, and microstructure between bioSiCs from different origins. Despite their variations, all biomorphic silicon carbide ceramics were able to load and release vancomycin. Oak bioSiC with the highest specific surface, the lowest total porosity, and the biggest open pores shows a slow vancomycin release rate that promotes an antibacterial effect for more than a week for materials including 2.54 mg of drug. Differences between materials in preventing *S aureus* biofilms are not found for already formed *S. aureus* biofilm treatments.

The internal structure and surface properties of all the systems seem to facilitate the therapeutic activity of the antibiotic on the preformed biofilm, reducing the viable amount of bacterial colonies with time, by maintaining drug release over MIC for a long period of time. The use of bioSiC loaded systems is a promising strategy not only to prevent postsurgical periprosthetic infections but also to treat already present infections.

## Figures and Tables

**Table 1:** BioSiC properties obtained by helium pycnometry, nitrogen adsorption and mercury intrusion porosimetry. Standard deviation in parentheses.

Sample	Density (g/cm^3^)	HG	Specific surface (m^2^/g)	HG	Porosity (%)	HG
Pine bioSiC	3.01 (0.01)	X	0.83 (0.05)	X	46.97 (5.43)	X
Oak bioSiC	2.90 (0.01)	X	1.10 (0.02)	X	27.85 (2.99)	X
Sapelli bioSiC	3.05 (0.01)	X	0.99 (0.05)	X	40.72 (1.06)	X

HG means homogeneous groups.

**Table 2:** The release kinetics of vancomycin of loaded bioSiCs (Dose = 1.27 mg/mL) by Higuchi model (*M* = *K*_*H*_∗*t* ^0.5^). Standard deviation in parentheses.

Sample	*K*_*H*_	*r* ^2^	*F*	Freedom degrees	*α*
Oak bioSiC	0.32 (0.07)	>0.90	>70	1 and 7	<0.01
Pine bioSiC	0.54 (0.15)	>0.94	>90	1 and 7	<0.01
Sapelli bioSiC	0.59 (0.06)	>0.86	>36	1 and 7	<0.01

*K*_*H*_ is the Higuchi release rate constant, *r* ^2^ is the square of the correlation coefficient, *F* is the *F*-ratio from the ANOVA of the regression and *α* is the probability of error.

**Table 3:** Number of CFUs of *S. aureus* on the biofilm at preset times (dilution 10^4^).

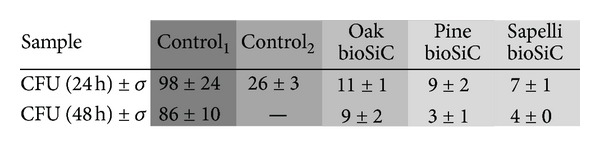

Differences in colour show statistically significant differences.
